# Editorial: Genes and Aging: From Bench-to-Bedside

**DOI:** 10.3389/fnagi.2022.886967

**Published:** 2022-03-30

**Authors:** Wael M. Y. Mohamed, Chenju Yi, Lilach Soreq, Toshihide Yamashita

**Affiliations:** ^1^Department of Basic Medical Science, International Islamic University Malaysia, Selayang, Malaysia; ^2^Department of Clinical Pharmacology, Menoufia University, Shebeen El-Kom, Egypt; ^3^Seventh Affiliated Hospital, Sun Yat-sen University, Shenzhen, China; ^4^UCL Institute of Neurology, University College London (UCL), London, United Kingdom; ^5^Department of Molecular Neuroscience, Osaka University, Suita, Japan

**Keywords:** genes, aging, neurodegeneration, dementia, AD

Aging is inevitable, and several structural and functional changes occur in the body with advanced aging. Aging affects the brain in a variety of ways, the most obvious of which are changes in motor, sensory, and cognitive function (Jeromin and Bowser, 2017; Ibrahim et al., 2020). Aging has becoming a remarkable public health issue worldwide: by 2050, the proportion of the world's population over 60 years will reach 22%. This increase in the number of populations aging calls for attention to aging-associated diseases, including Neurodegenerative disorders such as Alzheimer's Disease (AD) and Parkinson's Disease (PD) (Gazzaley et al., 2005). In the brain, different cell types, including neurons, astrocytes, oligodendrocytes, as well as microglia, respond to aging differently (von Bartheld et al., 2016; Chapman and Hill, 2020). Studying the transcriptome changes in different cell types during aging, and identification of cell type-specific aging markers would enable us to move the therapeutic window to earlier stages of aging-associated disease progression/premature aging. In addition, understanding the mechanism of how cell-type specific aging genes are involved in the aging process could further shed light on the prevention and therapy of the aging-related diseases (Soreq et al., 2017, 2021).

The present article collection aims at giving a reader the most up-to-date perspective on how the interaction between genes and neuropsychological processes leads to neurological disorders in normal aging and minor and major neurocognitive disorders. It is intended to provide an opportunity for researchers of different perspectives to discuss recent progress in this field. This volume aimed to provide an overview of contributions from basic, and clinical aspects to understand the interplay between genes and aging from Bench-to-Bedside. The authors from different research fields identified numerous existing and emerging genes/aging related topics that provide new and unique perspectives.

In this volume, several articles focus on Alzheimer's Disease. For instance, a review by Abubakar et al. discussed the updates on pathophysiology of Alzheimer's Disease that introduces a better understanding of novel signaling pathways associated with neural and glial mechanisms involved in AD, elaborates potential links between vascular dysfunction and AD, and recent developments in “omics”-based biomarkers in AD. Also, Sabaie et al. carried out a scoping review according to sic-stage methodology structure and PRISMA guideline to analyse validated loops of competing endogenous (ce)RNA in AD and focus on ceRNA axes associated with lncRNA with the therapeutic potential in AD. In parallel with this, Abuelezz et al. presented an updated analysis of miRNAs role in regulating signaling processes that are involved in AD-related pathologies. They discussed the current challenges against wider use of miRNAs and the future promising capabilities of miRNAs as diagnostic and therapeutic means for better management of AD. Further, this volume discussed AD dementia from different perspectives. Liu L et al. investigated the frequency of Chinese Han patients harboring genetic Frontotemproal Dementia (FTD) variants. They concluded that there was a high prevalence of genetic variants in Chinese bvFTD patients, highlighting the necessity of genetic testing for bvFTD. Similarly, Sun et al. analyzed the correlations of genetic features with clinical symptoms in patients with degenerative dementia. They recruited a group of 84 Chinese dementia patients and conducted the whole exome sequencing (WES). The data were analyzed focusing on 153 dementia-related causing and susceptible genes. They concluded that the new variants in dementia-related genes indicated heterogeneity in pathogenesis and phenotype of degenerative dementia. WES could serve as an efficient diagnostic tool for detecting intractable dementia. It is worth mentioning that the current volume contains a basic study conducted by Ba et al. they modeled chronic sleep fragmentation (SF) in young wild-type mice and detected pathological hyperphosphorylated-tau (Ser396/Tau5) and gliosis in the SF hippocampus. In summary, 1.5-month sleep fragmentation could generate AD-like pathological changes including tauopathy and gliosis, mainly linked to stress, as the incremented glucose metabolism observed with PET imaging suggested. Thus SF could eventually lead to chronic neurodegeneration if the stress condition is prolonged in time. This is supported by Zhang et al. as they summarized present knowledge about dynamic changes of oligodendroglial lineage cells during normal aging and discussed their potential roles in age-related functional decline. Especially, they focused on declined myelinogenesis during aging and underlying mechanisms. Their article was clarifying those oligodendroglial (OLG) changes and their effects on neurofunctional decline may provide new insights in understanding aging associated brain. Jin et al. examined the associations of four single nucleotide polymorphisms (SNPs) on aldehyde dehydrogenase (*ALDH*) and alcohol dehydrogenase (*ADH*) genes (i.e., *ALDH2* rs671, *ADH1B* rs1229984, *ADH1B* rs1042026, and *ADH1C* rs1693482) and cognitive impairment among the oldest-old. They examined data from the Chinese Longitudinal Healthy Longevity Survey genetic sub-study, including 1,949 participants aged over 90 years. They did not observe a significant interaction between those SNPs and alcohol consumption. Finally, Liu Y-H et al. discussed a case report leukoencephalopathy syndrome and they identified a novel homozygous mutation (NM_012268.3: c.186C>G/ p.Y62X) of *PLD3* in a consanguineous family with white matter lesions, hearing and vision loss, and kidney disease by whole exome sequencing. This may be the first case report on the homozygous mutation of PLD3 in patients worldwide.

Overall, this volume provides a rare opportunity to promote awareness and creativity for neurological disorders using animal models of different species, while stimulating testable theories and establishing a strategic research agenda to facilitate their integration into clinical studies.

## Author Contributions

WM wrote the original draft and incorporated suggestions from the co-authors. LS edited the paper draft. The other co-authors edited the manuscript. All authors contributed to the article and approved the submitted version.

## Funding

LS was funded by Rose Trees UK.

## Conflict of Interest

The authors declare that the research was conducted in the absence of any commercial or financial relationships that could be construed as a potential conflict of interest.

## Publisher's Note

All claims expressed in this article are solely those of the authors and do not necessarily represent those of their affiliated organizations, or those of the publisher, the editors and the reviewers. Any product that may be evaluated in this article, or claim that may be made by its manufacturer, is not guaranteed or endorsed by the publisher.
